# Human Radiosensitivity and Radiosusceptibility: What Are the Differences?

**DOI:** 10.3390/ijms22137158

**Published:** 2021-07-02

**Authors:** Laura El-Nachef, Joelle Al-Choboq, Juliette Restier-Verlet, Adeline Granzotto, Elise Berthel, Laurène Sonzogni, Mélanie L. Ferlazzo, Audrey Bouchet, Pierre Leblond, Patrick Combemale, Stéphane Pinson, Michel Bourguignon, Nicolas Foray

**Affiliations:** 1 Inserm, U1296 unit, Radiation: Defense, Health and Environment, Centre Léon-Bérard, 28, rue Laennec, 69008 Lyon, France; Laura.El-Nachef@inserm.fr (L.E.-N.); joelle.al-choboq@inserm.fr (J.A.-C.); Juliette.Restier--Verlet@inserm.fr (J.R.-V.); adeline.granzotto@inserm.fr (A.G.); elise.berthel@inserm.fr (E.B.); Laurene.sonzogni@inserm.fr (L.S.); melanie.ferlazzo@inserm.fr (M.L.F.); audrey.bouchet@inserm.fr (A.B.); Michel.Bourguignon@inserm.fr (M.B.); 2 Neolys Diagnostics, 67960 Entzheim, France; 3 Centre Léon-Bérard, 28, rue Laennec, 69008 Lyon, France; Pierre.Leblond@lyon.unicancer.fr (P.L.); Patrick.Combemale@lyon.unicancer.fr (P.C.); 4 Hospices Civils de Lyon, Quai des Célestins, 69002 Lyon, France; Stephane.pinson@lyon.unicancer.fr; 5 Université Paris Saclay Versailles St Quentin en Yvelines, 78035 Versailles, France

**Keywords:** radiosensitivity, radiosusceptibility, radiodegeneration, ATM, ionizing radiation

## Abstract

The individual response to ionizing radiation (IR) raises a number of medical, scientific, and societal issues. While the term “radiosensitivity” was used by the pioneers at the beginning of the 20st century to describe only the radiation-induced adverse tissue reactions related to cell death, a confusion emerged in the literature from the 1930s, as “radiosensitivity” was indifferently used to describe the toxic, cancerous, or aging effect of IR. In parallel, the predisposition to radiation-induced adverse tissue reactions (radiosensitivity), notably observed after radiotherapy appears to be caused by different mechanisms than those linked to predisposition to radiation-induced cancer (radiosusceptibility). This review aims to document these differences in order to better estimate the different radiation-induced risks. It reveals that there are very few syndromes associated with the loss of biological functions involved directly in DNA damage recognition and repair as their role is absolutely necessary for cell viability. By contrast, some cytoplasmic proteins whose functions are independent of genome surveillance may also act as phosphorylation substrates of the ATM protein to regulate the molecular response to IR. The role of the ATM protein may help classify the genetic syndromes associated with radiosensitivity and/or radiosusceptibility.

## 1. Introduction

The individual response to ionizing radiation has been the subject of a plethora of studies in the last decades, notably to evaluate the related risks, not only for exposed humans, but also for ecosystems. In particular, the International Commission of Radiation Protection (ICRP) set up in 2018 a dedicated group (TG111) to address the corresponding issues. Unfortunately, while the individual response to radiation is more and more documented, some of its various features are described with non-univocal terms, which does not facilitate their understanding and the elucidation of their intrinsic mechanisms.

### 1.1. Historical Features

The term ’radiosensitivity’ is one of the most extensively used words in radiation biology, oncology, and protection. The first occurrence of the “radiosensitivity” term was found in the French “radiosensibilité” and German “Strahlenempfindlichkeit” in 1907 [1]. The French “radiosensibilité” likely originates from “radioactivité” (radioactivity) that was proposed by Curie to replace the term “hyper-phosphorescence” initially chosen by Becquerel after his discovery of natural radioactivity [2]. Although the exact origin of this term is still unclear, “radiosensitivity” was systematically used to describe radiation-induced tissue reactions, such as skin burns, with the widely accepted hypothesis that these tissue events were associated with cellular death and that there was a causal link between clinical and cellular features [3]. This was notably the case of the reactions reported by Albers-Schönberg in 1898 in patients treated for lupus [4] and by Bouchacourt in patients treated for hypertrichosis [5]. 

By contrast, to describe the first radiation-induced cancers, notably that reported by Frieben in 1902 [6], and those from the over-exposed dial painters (the “radium girls”) between 1917 and 1926, the term “radiosensitivity” was not used but was simply replaced by “radiation-induced cancers” [7]. 

From the 1930s, English became the official language during the first International Congresses of Radiology, the term “radiosensitivity” was used indifferently whether for describing radiation-induced tissue reactions or cancers [8]. As a consequence, confusion has emerged [1].

### 1.2. A Current Confusion

Since the 1930s, in the ICRP publications, the term “radiosensitivity” was used as a synonym of:

- radiation-induced cancers, e.g., “Children are more radiosensitive than adults” [9] or “thyroid is a radiosensitive organ” [10];
- radiation-induced cataracts, e.g., “eyes are radiosensitive” [11];
- radiation-induced toxicity as adverse tissues reactions in “ataxia telangiectasia, caused by *ATM* mutations, is the most radiosensitive syndrome” [12].

A practical consequence of such confusion is to allow the belief that a “radiosensitive” patient may have the same quantitative risk of radiation-induced cancers, radiation-induced cataracts, and post-radiotherapy adverse tissue reactions. Additionally, such confusion may raise obvious legal issues, as radiation-induced cancers, cataracts, or skin burns do not obey the same incidence laws and do not correspond to the same level of clinical injuries and repairability [13]. 

### 1.3. The Evidence of a Molecular Difference

To date, while the term “radiosensitivity” is still used indifferently, there is increasing evidence that the molecular and cellular bases that lead to radiation-induced cellular death and tissue reactions are different from those that lead to radiation-induced and spontaneous cancer proneness. A typical example is given by three genetic syndromes:

- the Li Fraumeni’s syndrome (LFS), caused by the heterozygous mutations of *p53* [14] is associated with cancer proneness but not with significant post-radiotherapy adverse tissue reactions [14];
- the ataxia telangiectasia (AT) caused by the homozygous mutations of *ATM* [15] is associated with post-radiotherapy fatal reactions and AT patients are at high risk of leukemia [15,16];
- the Cockayne’s syndrome (CS) caused by the homozygous mutations of the *CS* genes [17] is associated with a significant tissue radiosensitivity but no cancer proneness [17,18].

Altogether, these observations strongly suggest that radiation-induced tissue reactions are not necessarily linked to spontaneous and radiation-induced cancer proneness. Practically, such a conclusion is important for radiation oncologists who should, in the first case (tissue radiosensitivity but not radiation-induced cancer proneness), decrease the total dose in the planned treatment, or even forbid any radiotherapy [16] while, in the second case (radiation-induced cancer proneness but not tissue radiosensitivity), they can treat their patient by considering his age: if the patient is young, they should reduce drastically the dose on healthy tissues surrounding the tumors, maybe by using new-generation radiotherapy modality, while if the patient is old, they may consider that the transformation of cells into a radiation-induced malignancy may be longer than the general life span of the patient. 

Similar conclusions can be reached with radiation-induced cataracts vs. tissue reactions and/or cancer proneness. For example, some genetic syndromes may be associated with both juvenile cataracts and tissue radiosensitivity like in the case of the Rothmund–Thomson syndrome [19], or else both juvenile cataracts and cancer proneness like in the case of the neurofibromatosis type 2 [20].

### 1.4. Univocal Definitions

To avoid all these confusions with the use of the term “radiosensitivity”, two approaches, at least, were possible:

- the “genomic approach” which consists of inventorying all the genes involved by their expression or polymorphisms in the high throughput studies of radiosensitivity, in order to establish causal links with clinical features [21]. The major advantage of this approach is to get a large number of candidate genes. The major inconvenience is to consider gene expression as a major feature of the response to radiation, while some cases of radiosensitivity are not necessarily linked to a higher or lower gene expression but to protein dysfunction [8];
- the “clinical approach” which consists of defining the major clinical features of the response to radiation, and thereafter to identify genes in each category. The major advantage of this approach is to gather all the different types of radiation-induced events observed by clinicians. The major inconvenience is to omit some genes that may be involved in the response to radiation while their mutations lead to non-viability and therefore are not associated with any described syndrome [8].

In the present review, we have considered only this second approach, arguing that it permits to alleviate the major confusions caused by non-univocal definitions of the terms used. In addition, this approach permits us to indifferently apply the following terms to the individual, tissue, cellular, or molecular scales. The following definitions have therefore been proposed in the literature:

- The radiosensitivity is the proneness to the adverse tissue events that are considered as non-cancer radiation-induced effects and attributable to cell death. Radiosensitivity is generally correlated with unrepaired DNA damage [12];
- The radiosusceptibility is the proneness to the radiation-induced cancers that are non-toxic radiation-induced effects attributable to cell transformation and genomic instability. Radiosusceptibility is generally correlated with misrepaired DNA damage [22]. As IR is considered as a carcinogenic agent, radiosusceptibility is strongly linked to susceptibility to spontaneous cancers. The term “radiosusceptibility” was proposed through its similarities with “cancer susceptibility”, extensively used in the ICRP reports, and as it introduces the notions of stochastic events [8];
- The radiodegeneration responses are non-cancer effects attributable to mechanisms related to accelerated aging. Radiodegeneration should be correlated with unrepaired DNA damage that is tolerated by and can cumulate in cells [8]. Radiodegeneration responses cannot be considered similar to radiosensitivity responses as defined above, as their incidence rates, the types of cellular death, and the genes involved are different.

The objective of this review is to better understand what the differences are between human radiosensitivity and radiosusceptibility and, therefore, to document the differences between the risks of radiation-induced adverse tissues and the risks of radiation-induced cancers.

## 2. The Different Features of Human Radiosensitivity

### 2.1. What do We Learn from the Quantification of Human Radiosensitivity?

In order to provide a complete classification of the human radiosensitive syndromes, it was important to quantify radiosensitivity by investigating the tissue, cellular, and molecular responses. 

At the tissue scale, the enormous amount of radio-pathological data led to the definition of consensual grading scales reflecting the severity of radiation-induced reactions whatever the organ of the tissue considered [8,23]. This is the case of the Common Terminology Criteria for Adverse Events (CTCAE) [24] and the Radiation Therapy Oncology Group (RTOG) [25] scales. These two scales classify radiation-induced tissue reactions in 6 grades (grade 0: no event; grade 5: death), for each organ. To date, the CTCAE or RTOG severity grades are still the most reliable endpoints to quantify the clinical radiosensitivity [12].

At the cellular scale, radiosensitivity corresponds to an excess of cellular death. One of the most common assays to quantify radiation-induced cell death is based on the clonogenicity of irradiated cells: after irradiation, each clonogenic cell that provides daughter cells forming a colony is considered as surviving [26]. The clonogenic cell survival obeys the empirical linear-quadratic (LQ) model [27,28,29]. The surviving cell at 2 Gy (SF2; 2 Gy corresponds to the average dose par standard radiotherapy session) is one of the most reliable parameters for quantifying cellular radiosensitivity. SF2 was found to be correlated with the in vivo radio-responsiveness [30].

At the cytogenetic scale, radiosensitivity has long been correlated with the number of micronuclei [31] and unrepaired chromosome breaks [32]. It is noteworthy that Cornforth and Bedford have shown that one single unrepaired chromosome break corresponds to a lethal event for the cell [33]. 

At the molecular scale, the prediction of radiosensitivity is considered as the “Holy Grail” of radiobiology. This quest has led to the development of molecular predictive assays that are designed to be faster than the clonogenic cell survival assay described above. 

### 2.2. Genetic Syndromes Associated with Radiosensitivity from the Clinical, Cellular and Molecular Criteria

By considering the post-radiotherapy (clinical) tissue reactions as a criterion, only four genetic syndromes have been formerly associated with radiosensitivity through the observation of fatal reactions (grade 5) (which may be called hyper-radiosensitivity): ataxia telangiectasia (AT) (homozygous *ATM* mutations), LIG4 syndrome (homozygous *LIG4* mutations), Nijmegen’s syndrome (homozygous *NBS1* mutations), and a variant of Xeroderma Pigmentosum C (homozygous *XPC* mutations):

- AT patients show a very high risk of leukemia/lymphoma, and were treated by radiotherapy (total body irradiations) in the 1970s [16,34,35,36]. The severity of their post-radiotherapy reactions (nearly all fatal) and their extreme sensitivity to other DSB-inducing drugs have imposed a particular care for treating them with radiomimetic drugs [16,37,38,39]. AT is caused by homozygous or compound heterozygous mutations of the *ATM* gene [40,41];
- LIG4 syndrome was first described from an acute lymphoblastic leukemia patient who overresponded to radiation therapy and died following radiation morbidity, without showing any clinical features in common with AT. The radiobiological characterization of his cells revealed homozygous mutation of Ligase IV [42,43,44,45];
- some patients suffering from Nijmegen’s syndrome, such as the notable case of a young patient with medulloblastoma, showed severe toxicity to radio- and chemotherapy [46,47,48]. Nijmegen’s syndrome is caused by homozygous mutations of the *NBS1* gene [47,49];
- a case of a Xeroderma Pigmentosum C patient who suffered from an angiosarcoma and showed a fatal reaction to radiotherapy was reported [50]. However, the radiosensitivity of this case has been shown to be complemented by the transfer of a wild-type chromosome 8, suggesting that this XPC variant patient may hold other gene mutation responsible of his abnormal response to radiation [51,52].

In addition to the clinical hyper-radiosensitivity revealed by fatal post-radiotherapy reactions, some cases of patients suffering from Bloom’s syndrome [53] and Fanconi Anemia [54] associated with severe to moderate post-radiotherapy tissue reactions (CTCAE grade 2–4) have been reported. Interestingly, as these cases of radiosensitivity have been pointed out during or after an anti-cancer radiotherapy, they suggest a strong link between high cancer proneness and hyper-radiosensitivity. However, when clonogenic cell survival and cytogenetics criteria are considered, the picture changes, and some other syndromes associated with radiosensitivity, but not with a high risk of cancer, do appear (Table 1). Indeed, between the 1970s and the 1990s, a plethora of genetic syndromes have been characterized radiobiologically, and the first surveys of human radiosensitivity were reported [55,56,57]. Hence, while ataxia telangiectasia, LIG4 syndrome, Nijmegen’s syndrome, and certain XPC cases elicit the lowest SF2 values and the most severe cytogenetic data, progeroid [58], Werner’s [59], Usher’s [60], and Cockayne’s syndromes [17], i.e., a subset representative of aging and cellular degeneration, appear to be associated with very severe cellular radiosensitivity, but not with a high risk of cancer (Table 1). It must be stressed that the radiosensitivity associated with these syndromes was found in vitro, as the corresponding patients were not exposed to any radiation treatment. Consequently, these genes, although involved in the human response to radiation, do not appear in the genomic approaches [21].

**Figure 1:**
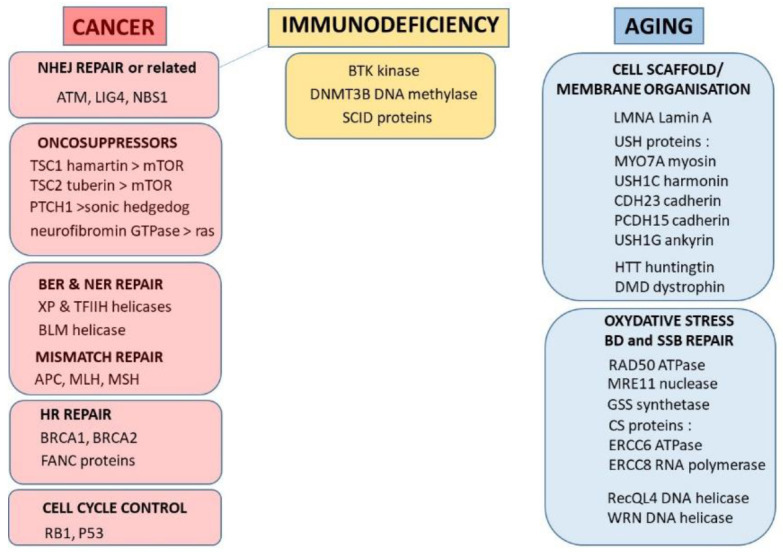
Summary of the major radiosensitive syndromes described in Table 1 and represented by their proteins and their biological role in the cell. There is still no data available to classify radiosensitive syndromes caused by *BTK*, *DNMT3B*, and *SCID* mutations as associated with aging or cancer proneness similar to *ATM*, *LIG4*, and *NBS1* mutations.

**Figure 2:**
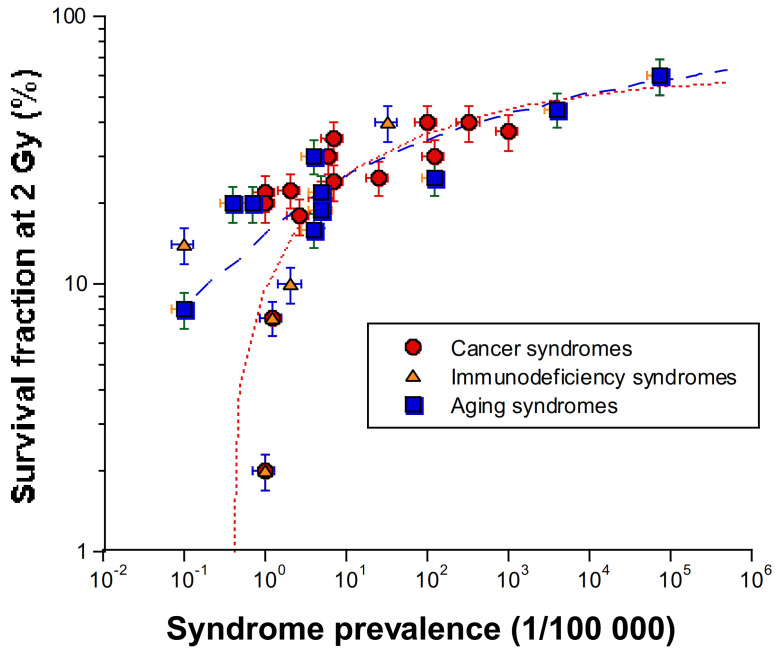
Relationship between radiosensitivity (represented by SF2) and prevalence for the syndromes described in Table 1. Syndromes with only few cases were omitted. Dotted lines represent a data fit to a sigmoidal formula.

A further analysis of the human radiosensitivity data of Table 1 leads to two important conclusions:

- some radiosensitive diseases (reviewed in Table 1), appear to be caused by mutations of genes that encode cytoplasmic proteins, suggesting that radiosensitivity may not be due to impaired events occurring only in the nucleus [61] (Figure 1);
- as SF2 increases, the syndromes caused by homozygous mutations (leading to loss of functions) are progressively replaced by syndromes caused by heterozygous mutations (leading to “leaky” functions) (Table 1). Syndromes caused by heterozygous mutations being more frequent than those caused by homozygous mutations, *SF2 increases with prevalence* (Figure 2).

By considering the molecular point of view, and by hypothesizing impairments in DSB repair and signaling pathways are the major causes of human radiosensitivity, the mutations of the major DSB repair genes should provide the highest radiosensitivity features. In humans, two major DSB repair pathways have been pointed out: the non-homologous end-joining (NHEJ), predominant in quiescent cells [62], and the homologous recombination (HR), predominant in proliferating cells [63]. Interestingly, the homozygous mutations of the major NHEJ and HR genes lead to embryonic lethality in humans: this is notably the case of *Ku*, *DNA-PKcs*, *RAD51*, *RAD52*, *BRCA1*, and *BRCA2* genes [8]. The most relevant explanation of this observation is that very few (i.e., one or two) unrepaired DSB lead to cell lethality [12,22,33]: the functions of these genes are so important for cellular viability and the first mitoses that their loss results in early embryonic lethality. The only major DSB repair gene whose homozygous mutations correspond to a viable syndrome is *LIG4*. Conversely, the fact that some homozygous *LIG4* mutations caused an existing syndrome indicates that the Ligase IV protein may be dispensable for certain steps of NHEJ pathway [64]. The role of some other NHEJ genes have been pointed out. This is notably the case of *Artemis*, *XLF/Cernunnos*, and *RAG2/RAG2* genes that are also involved in V(D)J recombination [65]. However, it is noteworthy that the corresponding severe combined immunodeficiency diseases (SCID), Artemis, Cernunnos, and Omenn’s syndromes do not show a marked radiosensitivity, suggesting again that these corresponding genes play dispensable roles in the different NHEJ steps [66].

With regard to the mutations of the genes involved in other DNA repair pathways, their link to radiosensitivity may be explained either by their relative impact in DSB repair pathways or by the importance of the DNA damage other than DSB on IR cell death. For example, Table 1 and Figure 1 reveal that syndromes associated with mutations of the secondary HR proteins such as BRCA1 and BRCA2 do exist, but their radiosensitivity is not severe, which can be explained by the fact that HR is not a predominant DSB repair pathway in humans, and likely concerns the minority of proliferative cells of the body. With regard to the moderate but significant radiosensitivity observed with the AT-like disorder (ATLD) (*MRE11* mutations), Fanconi anemia (*FANC* genes mutations), Bloom’s syndrome (*BLM* mutations), and xeroderma pigmentosum (*XP* genes mutations), it must be stressed that all these mutations affect the repair of DNA mismatch (DM), base damage (BD) or DNA single-strand breaks (SSB) that may be involved in the cell death or interplay with DSB repair pathways.

Interestingly, our review highlights some cases of significant radiosensitivity with mutations of genes whose function was not expected in the radiation response. This is notably the case of progeroid syndromes [58,67,68], Usher’s syndrome [60,69], Bruton’s disease [70,71], and Huntington’s disease [72,73,74]. These syndromes are caused by mutations of genes that encode for cytoplasmic proteins, or are localized in the nuclear membrane and whose functions concern the cell scaffold and membrane organization. It is noteworthy that these genes are not detected by the genomic approach [21]. The presence of such disorders in the list of the radiosensitive syndromes will be discussed in the next chapters (Figure 1).

To conclude, the review of human radiosensitive syndromes shows that:

- homozygous mutations of *ATM*, *LIG4*, and *NBS1*, involved in the DSB repair and signalling pathways, cause the most hyper-radiosensitive syndromes; this hyper-radiosensitivity has been observed both clinically and in vitro;
- there is a continuum of SF2 values form 1% (ataxia telangiectasia) to about 60% (average radioresistance). Surprisingly, to the notable exceptions of the three precited syndromes, there are few syndromes caused by mutations of DSB repair proteins, probably as DSB is a key-DNA damage that impacts on each step of embryogenesis. About 50% of radiosensitive syndromes are caused by genes involved in the repair of radiation-induced DNA damage (other than DSB) that may affect cell survival after irradiation. The remaining 50% are caused by genes involved in the cell scaffold and the nuclear membrane, and whose encoded proteins are cytoplasmic (Figure 1).

## 3. The Different Features of Human Radiosusceptibility

### 3.1. What do We Learn from the Quantification of Human Radiosusceptibility?

As described in the Introduction, the first radiation-induced cancers was reported by Frieben on his own hand (1902) and throughout the cohort of the radium girls [7]. However, the first dose–response curve involving radiation-induced cancers was obtained from the studies of atomic-bomb survivors of Hiroshima and Nagazaki [75]. Thereafter, a number of epidemiological reports have described a link between cancers and radiation doses, notably from nuclear workers of the former USSR, miners exposed to radon, and ankylosing spondylitis or tuberculosis patients treated by IR [76]. There are four major conclusions that can be reached from these databases:

- these cohorts/databases, derived from epidemiological data, do not highlight any individual predispositions to specific malignancies, but concern a whole population of individuals considered as equally radioresistant. The dose-effect curve shape may vary according to the type of radiation-induced cancer;
- there is no clinical equivalent of CTCAE/RTOG scales grading the different steps of carcinogenesis. Consequently, the relative risk (RR) or the excess of relative risk (ERR) are the only parameters to express cancer incidence as a function of dose. It is noteworthy that these parameters are calculated from epidemiological data [8];
- there is no consensual mathematical model that describes (similar to the LQ model for cell survival) the cancer incidence, or its risk as a function dose. Indeed, the radiation-induced cancer incidence curves are generally described as either linear with no threshold (LNT) or non-linear with a threshold (NLT). The LNT/NLT controversies have long reflected a societal issue, raising the question of the existence of a dose-threshold below which there are no significant association between malignancies and exposures to ionizing radiation [77,78]. From Hiroshima survivors data, the threshold doses have been found to be 100 mGy for radiation-induced leukemia and 200 mGy for radiation-induced solid cancers [75]. However, these dose thresholds are relevant only for high dose-rate (flash) exposures to radiation. The corresponding dose thresholds for low dose-rate exposures are still unknown [79].

With regard to the molecular, cytogenetic, and cellular point of views, the quantification of cancer proneness is still made difficult by the uncertainties about the intrinsic mechanisms of carcinogenesis. While the characterization of hereditary retinoblastoma results in the concept of tumor suppressor genes [80], it is proposed, to date, that the cellular transformation from normal cells to neoplastic state obey three consecutive steps (initiation, promotion, and progression) with a progressive acquisition of some specific properties such as sustaining proliferative signaling and escaping genome integrity surveillance [81,82]. Hence, a cancer would result from a combination of misrepaired DNA lesions disturbing its homeostasis, where a minimum of 10 lesions seems necessary [83] or possibly less when one oncogene is activated [84]. Particularly, spontaneous and radiation-induced cancer proneness have been associated with the lack of control of the recombination process (hyper-recombination) [85,86,87]. Such a process may lead to misrepaired DSB, chromosome translocations, and high rates of mutations such as those observed with the hypoxanthine-guanine phosphoribosyltransferase (HPRT) assay [88]. Briefly, the HPRT assay consists of using the HPRT locus as a reporter gene and in identifying each mutant. HPRT data lead to a dose–effect relationship. Unfortunately, the HPRT assay has not been applied systematically for all the radiobiological characterization of genetic syndromes described in Table 1. This is also the case of cytogenetic assays, or any techniques based on the detection of misrepaired DNA damage. As the lack of control of cell cycle checkpoints is one of the most common features of cellular transformation, another approach based on the assessment of the impairment of G2/M arrest has been developed. The G2 assay that consists in assessing the efficiency of the G2/M checkpoint has been successfully applied to a number of cancer prone diseases [89,90,91]. Again, despite the high specificity of this assay, there is no systematic data available to characterize each syndrome described in Table 1. To conclude, the study of human radiosusceptibility still needs further investigations and systematic approaches to establish a classification that would help clinicians in their choice of treatment.

### 3.2. Genetic Syndromes Associated with Radiosusceptibility from the Clinical, Cellular, and Molecular Criteria

While there is no apparent correlation between the type of cancer and radiosensitivity, Table 1 shows that some radiosensitive syndromes may be associated with a large spectrum of malignancies This is notably the case of heterozygous *BRCA1/BRCA2* mutations that may lead to fallopian tube cancer, primary peritoneal cancer, female and male breast cancer, pancreatic cancer, and prostate cancer [92]. Hence, it appears difficult to consider one type of malignancy (e.g., leukemia) and to analyze its occurrence for each syndrome. Similarly, by taking the highest relative cancer risk associated to a given syndrome, whatever the type of cancer, no correlation with radiosensitivity appeared. A typical example of this statement is to consider the relative risk of leukemia associated with homozygous *ATM* mutations (which is significantly higher than 10) on one hand, and, on the other, the relative risk of breast cancer associated with heterozygous *BRCA1* mutations (between 2 and 10) that provides a very weak radiosensitivity [93]: there is no intermediate relative risk of cancer corresponding to intermediate radiosensitivity (Table 1). Hence, unlike SF2, the risk of cancer does not vary with prevalence (Figure 2). However, whether they are associated with cancer or aging, the radiosensitive syndromes obey the same relationship between SF2 and prevalences higher than 1/100,000. To explain the differences between aging and cancer syndromes with prevalence lower than 1/100,000, it can be hypothesized that misrepaired DSB at the origin of cancer syndrome may affect early cell viability less significantly than that the tolerated unrepaired DNA strand breaks at the origin of aging syndromes. Further investigations are needed to document better such hypothesis.

Another approach to characterize radiosusceptibility is to analyze the role of the proteins encoded by the genes described in Table 1. As expected, genes involved in the susceptibility of spontaneous and radiation-induced cancers are related to proto-oncogenes, or oncogenes such as tuberous sclerosis [94,95,96,97] and neurofibromatosis type 1 [98,99,100,101] and type 2 [102,103,104], or else to repair of DNA damage that may be lead to misrepair (Figure 1). Again, among these syndromes, some are caused by mutated cytoplasmic proteins. However, this can be explained by the subcellular localization to some oncogene proteins and transcription factors that are not necessarily nuclear (note that the proteins are synthetized in cytoplasm).

To conclude, the review of human radiosusceptibility shows that:

- homozygous mutations of *ATM*, *LIG4*, and *NBS1* genes are associated with high risks of leukaemia/lymphoma;
- there is no consensual parameter to quantify radiosusceptibility, notably as the intrinsic mechanisms of carcinogenesis are still unknown;
- the radiosensitive syndromes that are associated with radiosusceptibility may be associated with a large spectrum of malignancies for a single gene mutation;
- the radiosusceptible syndromes are caused by mutations of genes related to proto-oncogenes, to radiation-induced misrepaired DNA damage, or else to cell cycle checkpoints (Figure 1). Again, among these syndromes, some are caused by mutated cytoplasmic proteins.

## 4. Toward a Unified Model for Radiosensitivity and Radiosusceptibility

### 4.1. Biological Function of Proteins as Proteins or as Substrates?

The radiosensitivity associated with Huntington’s disease, Usher’s syndrome, Bruton’s disease, and other syndromes caused by the mutations of cytoplasmic proteins involved in the cell scaffold remains an enigma. Indeed, while a dose of 2 Gy X-rays is sufficient to kill more than 80% irradiated cells, the same dose cannot significantly modify the protein scaffold of cells, as a significant radiation-induced modification of proteins would require much higher doses [105]. Similarly, a number of proteins evoked in Table 1 become cytoplasmic when mutated, and generally overexpressed. This is notably the case of RB1 [106], TP53 [107], and neurofibromin (Combemale et al., submitted). In addition, the function of these proteins seems not to vary with radiation dose, while their abundancy increases with it in cytoplasm. A last example is given by a recent study of radiosensitive variants of XPD, whose cells show cytoplasmic XPD forms while the function of the XPD protein has long been considered as a (nuclear) DNA helicase: what would be the function of a DNA helicase in cytoplasm [108]? Hence, the response to radiation of cells derived from the syndromes caused by some cytoplasmic proteins cannot be entirely explained by the biological role of the mutated proteins itself. How to explain this statement? A given protein may be characterized by its biological function that can be different in nucleus or in cytoplasm, but also by its role *as substrate* of some other proteins such as kinases.

Interestingly, the radiation response involves a number of proteins serving as substrates of phosphorylation, a very current radiation-induced biochemical transformation of proteins. Additionally, among the plethora of kinases that are responsible for radiation-induced phosphorylation, the ATM protein kinase has been considered as an early actor of the DSB recognition via its phosphorylation of H2AX, and the individual radiation response [69,70]. ATM has not been integrated *directly* in the repair steps of any DSB repair pathway models, while the homozygous *ATM* mutations result in the highest hyper-radiosensitivity observed in humans (Table 1). This statement suggests that radiosensitivity is not necessarily caused by a gross DSB repair defect, but can be also explained by a lack of DSB recognition, upstream of the DSB repair steps. The ATM protein is a serine/threonine kinase that phosphorylates a number of protein substrates holding SQ/TQ domains [109]. It was shown that ATM participates to a series of kinetically ordered stress response steps such as DNA damage recognition, repair, cell cycle checkpoint, and cellular death [110].

### 4.2. The Nucleo-Shuttling of ATM as a Primum Movens of the Molecular Response to Radiation

Recently, the delay in the radiation-induced nucleo-shuttling of the ATM protein (RIANS) was shown to be a reliable parameter for predicting radiosensitivity [12,111,112,113], and to provide a biologically relevant interpretation of the linear-quadratic model, the mathematical basis of the cellular radiation response [114]. In the context of the RIANS model, the following kinetically ordered steps have been observed:

- the “dosimetry step”: after irradiation, the production of reactive oxygen species (ROS) is dose-dependent. Under the effect of the radiation-induced oxidative stress, some cytoplasmic dimeric forms of ATM become monomeric in a dose-dependent manner;
- the “diffusion step”: the ATM monomers diffuse to the nucleus; however, during their course from the cytoplasm to the nucleus, they can meet some cytoplasmic ATM substrates with which they can form multiprotein complexes that prevents the nucleo-shutting;
- the “recognition step”: the remaining free ATM monomers diffuse to the nucleus and phosphorylate H2AX molecules at DSB sites, which activates NHEJ. The ATM monomers will re-dimerize during the DSB repair process and can be easily quantifiable as nuclear foci by immunofluorescence [61].

Interestingly, nearly all the proteins cited in Table 1 are known to be ATM phosphorylation substrates or hold SQ/TQ domains. A great majority of these proteins show cytoplasmic forms, or become cytoplasmic when mutated, suggesting that nearly all the radiosensitive syndromes described in Table 1 may potentially reach the requirements to be integrated in a unified mechanistic model based on the RIANS. ATM is not an exception to the RIANS model, as the hyper-radiosensitivity given by the homozygous *ATM* mutations can be interpreted as a dramatic lack of recognition of the radiation-induced DSB, leading to the absence of their repair by NHEJ [61]. Conversely, the hyper-radiosensitivity linked to the LIG4 syndrome is simply due to the loss of ligase IV function: while the RIANS appears to be normal in the *LIG4-*mutated cells, the ligase IV may not serve as substrate in cytoplasm, as the ligase IV protein remains in the nucleus even after the irradiation [12,22]. Hence, the homozygous *LIG4* mutations cause a gross DSB repair defect in the frame of the NHEJ pathway. Considering that the mutations of all the other DSB repair proteins upstream LIG4 are lethal, the LIG4 syndrome might be the only viable radiosensitive human syndrome directly caused by the loss of the function of its associated protein. Conversely, the radiosensitivity associated with all the other radiosensitive syndromes described in Table 1 may be caused, at least partially, by the role of the mutated proteins as ATM phosphorylation substrates in cytoplasm. Indeed, it has been shown that any delay of the RIANS leads to a phenotype of radiosensitivity via over-expressed ATM substrates called “X-proteins” [61]. If the diffusion of ATM monomers to the nucleus is delayed, DSB will not be recognized by NHEJ via the ATM-dependent phosphorylation of H2AX histone. Thereafter, three scenarios are possible:

- DSB are not repaired, whatever the repair pathway: these DSB become lethal and lead to radiosensitivity. Less than two unrepaired DSB are sufficient to cause cell death in humans;
- DSB are not recognized by NHEJ, but they are managed by a rapid but illegitimate hyper-recombination process: these DSB become misrepaired and lead to radiosusceptibility; they can be accompanied by additional DNA strand breaks due to hyper-recombination early after irradiation;
- DSB are tolerated (i.e., non-lethal immediately, likely as a longer cellular death process such as senescence rather than mitotic death or apoptosis). Progressively with time, the number of these DSB and SSB cumulate in cells to give a late subset of additional DNA damage.

**Figure 3:**
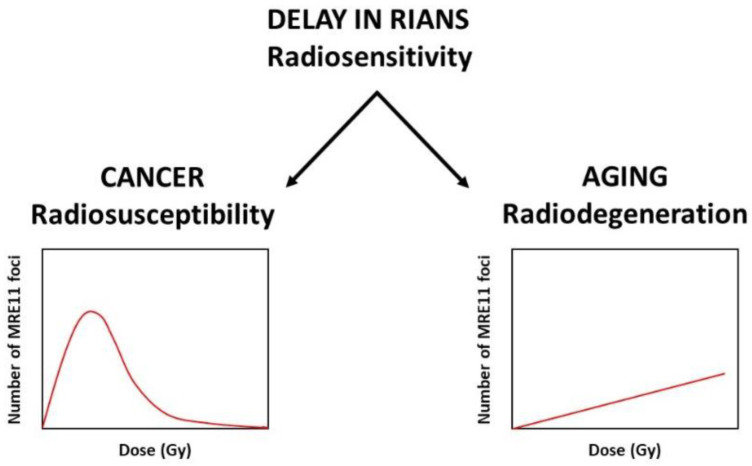
Relationships between radiosensitivity, radiosusceptibility, and radiodegeneration throughout the RIANS model and the MRE11 foci kinetics.

In response to ionizing radiation, the MRE11 nuclease relocalizes as nuclear foci, whose kinetics may significantly differ according to cell type and status [22]. Interestingly, the number of MRE11 foci observed after irradiation show an early (1 h after 2 Gy) increase in *RB1*-, *p53*-, *TSC*-, and *NF1*-mutated cells, and the MRE11 foci are impaired in *BRCA1*-, *BRCA2*-, *BLM*-, *FANC*-, and *ATM*-mutated cells (i.e., derived from mostly cancer syndromes) [61,106]. Conversely, the number of MRE11 foci progressively increases to reach its maximum 24 h after 2 Gy for *LMNA*-, *HTT*-, and *USH*-mutated cells (i.e., derived from mostly aging syndromes) (papers submitted). Once in nucleus, ATM may inhibit the MRE11 nuclease activity through its phosphorylation, which triggers the formation of nuclear foci: it is noteworthy that MRE11 foci are *inactivation* foci, unlike the γH2AX and pATM ones. Hence, our data suggest that cancer and aging syndromes may be characterized by the MRE11 foci kinetics [61]. However, further investigations are needed to better document the MRE11 data in cells derived from all the radiosensitive syndromes described in Table 1 (Figure 3).

## 5. Conclusions

The notions of radiosensitivity, radiosusceptibility, and radiodegeneration appear to be necessary to better describe the different types of individual radiation responses, and permit an evaluation of the specific radiation-induced risks in agreement with clinical observations. This review reveals that there are very few syndromes associated with the loss of biological functions involved directly in DNA damage recognition and repair bas their role is absolutely necessary for cell viability. Interestingly, some cytoplasmic proteins, whose functions are clearly different from genome surveillance, may also act as ATM phosphorylation substrates to regulate the DSB recognition and repair. The RIANS model may therefore provide a novel approach to classify the genetic syndromes associated with abnormal individual response to radiation, whether linked to toxicity, cancer, or aging.

## Figures and Tables

**Table 1:** The major human syndromes associated with radiosensitivity and/or radiosusceptibility.

Syndromes	Mutated Genes	Major Defective Mechanism	Prevalence per 100,000	SF2 (%)	Cancer Predisposition	Aging Neurodegeneration	Immuno- Deficiency	Subcellular Localization of the Protein
Ataxia telangiectasia	Homoz *ATM* mutations	DSB signaling and repair	~1	1–5	Leukemia, Lymphoma	No	Yes	Nucleus Cytoplasm
Ligase IV syndrome	Homoz *LIG4* mutations	NHEJ	Few cases	2–6	Leukemia, Lymphoma	No	Yes	Nucleus
Nijmegen’s syndrome	Homoz *NBS1* mutations	DSB signaling and repair	~1	5–9	Leukemia, Lymphoma	No	Yes	Nucleus
Hutchinson-Gilford Progeria syndrome	Heteroz* LMNA mutations	Nuclear membrane	0.12–0.25	8–19	No	Yes	No?	Inner nuclear membrane
Agamma-globulinemia Bruton’s disease	X-linked homoz *BTK* mutations	V(D)J recombination	1.4–2.8	10	No Some cases of colorectal cancer due to infections	No	Yes	Nucleus Cytoplasm
**Hypogamma-globulinemia** **Lig I deficiency**	**compound** **heteroz *LIGI* mutations**	**NER**	**one case**	**11**	**No**	**No**	**Yes**	**Nucleus** **Golgi apparatus** **Vesicles**
ICF syndrome	*Homoz, compound heteroz, DNMT3B mutations*	DNA methylation	~50 cases	14	No?	Yes?	Yes	Nucleus but also cytoplasm in mutated cells
Glutathione synthetase deficiency	most compound heteroz *GSS* mutations	Glutathione cycle	~70 cases ~0.1	14	No	Cerebellar degeneration in some severe cases	No?	Nucleus
NBSLD Syndrome	Homoz, compound heteroz *RAD50* mutations		Few cases	15	No?	Yes?	No	Nucleus
ATLD Syndrome	Homoz or compound heteroz *MRE11* mutations		Few cases	15–30	No	Yes?	No	Nucleus Cytoplasm
Cockayne’s syndrome	Homoz or compound heteroz *CS* mutations	NER/TCR	0.4	15–30	No	Yes	No	Nucleus
Xeroderma pigmentosum	Homoz or compound heteroz *XP* mutations	NER/TCR	0.4 to 1	15–30	Skin cancer	Yes	No	Nucleus only, except for XPD (both nucleus and cytoplasm)
Usher’s syndrome	Homoz *USH* mutations		3–5	16	No	Yes?	No	Cytoplasm
Huntington’s disease	Heteroz (gain-of-function) *HTT* mutations	DNA methylation	4–7	19	No	Yes	No	Nucleus Cytoplasm
Duchesne’s dystrophy	X-linked *DMD* mutations		1–9	16–28	No	Yes	No	Cytoplasm
Fanconi Anemia	Homoz or heteroz X-linked *FANC (A to D)* mutations		1	15–40	Leukemia, squamous cell carcinoma Breast cancer	No	Yes	Nucleus only, except for FANCD both nucleus and cytoplasm
Bloom’s Syndrome	Homoz or compound heteroz *BLM* mutations	HR/TLS	0.5–2	15–40	leukemia, lymphoma	No	Yes	Nucleus Cytoplasm
Gorlin’s (NF2) syndrome	Heteroz or de novo *PTCH1* mutations		1–9	12–30	Non-melanoma skin cancer	No	No	Golgi apparatus Cytoplasm domains
Tuberous sclerosis Complex syndrome	Heteroz *TSC* mutations	DSB signaling and repair	4–10	24	CMS and PMS tumors	No	No	Cytoplasm
Von Recklinghausen (NF1) syndrome	Heteroz or de novo *NF1* mutations	DSB signaling and repair	200–300	15–35	CMS and PMS tumors	No	No	Nucleus Cytoplasm
Li-Fraumeni syndrome	Heteroz *p53* mutations	Cell cycle regulation	4–10	20-50	breast, brain, leukemia, sarcoma	No	No	Nucleus Cytoplasm
Gardner’s syndrome	Heteroz *APC* mutations	Cell adhesion	2.2–3.2	18–30	Mainly colorectal cancer	No	No	Nucleus Golgi apparatus
Turcot’s syndrome	Homoz, compound heteroz, heteroz *MLH* mutations	MMR	~150 cases	21–30	Mainly colorectal cancer	No	No	Nucleus
Hereditary retinoblastoma	Heteroz *RB1* mutations	Cell cycle regulation	5–7	25–35	Retinoblastoma, sarcoma, melanoma, lung and breast cancer	No	No	Nucleus but also cytoplasm in mutated cells
Hereditary breast/ovary cancer	Heteroz *BRCA2* mutations	HR	~125	20–40	Breast/ovary cancer	No	No	Nucleus Cytoplasm
Hereditary breast/ovary cancer	Heteroz *BRCA1* mutations	HR	~333	30–50	Breast/ovary cancer	No	No	Nucleus Cytoplasm
AT heterozygotes	Heteroz *ATM* mutations	DSB signaling and repair	1000	20–55	High risk of breast cancer	No	No	Nucleus Cytoplasm
Werner syndrome	Homoz or compound heteroz *WRN* mutations	HR/TLS	2.5–5	20–55	some rare cancers	Yes	No	Nuclear Cytoplasm for some mutations
Rothmund-Thomson syndrome	Homoz or compound heteroz *RecQL4*mutations	HR/TLS	~300 cases	30–50	osteosarcoma	Yes	No	Nucleus Cytoplasm
Severe combined immunodeficiency	Homoz or compound heteroz *Cernnunos or Artemis* mutations	V(D)J recombination NHEJ	~33	30–50	Some rare lymphoma	No	Yes	Nucleus
Down’s syndrome	Chromosome 21 trisomy		100–150	25	High risk of ALL and AML	Yes	Yes	-
Lynch’s syndrome	Heteroz *MLH1*, *MSH2/6*, *hPMS2* mutations	MMR	100–125	30–50	Mainly Colorectal cancer	No	No	Nucleus
Alzheimer’s disease			2000–4000		No?	Yes	No	-

Abbreviations: homoz, homozygous; heteroz, heterozygous; APC, adenomatous polyposis coli; AT, ataxia telangiectasia; ATM, ataxia telangiectasia mutated; BLM, Bloom; BRCA1/2, breast cancer susceptibility gene 1/2; BTK, Bruton’s tyrosine kinase; CS, Cockayne syndrome; DNMT3B, DNA methyltransferase 3B; FANC, Fanconi anemia; GSS, glutathione synthetase; HR, homologous recombination; ICF, immunodeficiency–centromeric instability–facial anomalies. IR, ionizing radiation; Lig, ligase; MMR, mismatch repair; hMLH1, human DNA mismatch repair 1; MRE11, meiotic recombination 11; NBS, Nijmegen breakage syndrome; NER, nucleotide excision repair; NF1, neurofibromatosis type I; NHEJ, non-homologous end joining; PTCH1, patched 1 gene; RB; retinoblasoma; RecQ; recombinase Q; RecQL4; recombinase Q-like 4; SF2, surviving fraction at 2 Gy; TCR, transcription coupled repair; TLS, translesion synthesis; TSC, tuberous sclerosis complex. V(D)J, variability, diversity, joining; WRN, Werner. XLF, X-ray repair cross complementing 4-like factor; and XP, xeroderma pigmentosum.
